# P-1109. A phase 2, randomized, double-blind study of ibezapolstat compared with vancomycin for the treatment of *Clostridioides difficile* infection: clinical and microbiome evaluation

**DOI:** 10.1093/ofid/ofae631.1297

**Published:** 2025-01-29

**Authors:** Taryn A Eubank, M Jahangir Alam, Khurshida Begum, Jacob McPherson, Jinhee Jo, Michael Silverman, Kevin W Garey

**Affiliations:** University of Houston College of Pharmacy, Houston, Texas; Department of Pharmacy Practice and Translational Research, University of Houston College of Pharmacy, Houston, Texas, USA, Houston, Texas; Department of Pharmacy Practice and Translational Research, University of Houston College of Pharmacy, Houston, Texas, USA, Houston, Texas; University of Houston College of Pharmacy, Houston, Texas; University of Houston, Houston, Texas; Acurx Pharmaceuticals, LLC, White Plains, NY; University of Houston, Houston, Texas

## Abstract

**Background:**

Ibezapolstat (IBZ) is Gram-positive selective spectrum antibiotic that inhibits the bacterial DNA polymerase IIIC currently in clinical trial development for the treatment of *C. difficile* infection (CDI) in adults. In the open-label, non-comparative, phase 2a study, 10 of 10 IBZ-treated CDI patients experienced clinical cure. The purpose of the phase 2b CDI study was to assess the safety, efficacy, and microbiome change of IBZ versus vancomycin (VAN).

**Methods:**

Phase 2b (ClinicalTrials.gov, number NCT04247542) was a randomized, double-blind, active-comparator study. Participants with signs and symptoms of CDI and a positive enzyme immunoassay toxin test result were recruited from 12 centers in the USA and randomly assigned (1:1) to receive oral IBZ 450 mg every 12 h or oral VAN 125 mg every 6 h for 10 days. Stool was collected daily for microbiome evaluations. The primary endpoints were clinical cure at the end of therapy visit and safety. Secondary endpoints included microbiologic and microbiome evaluations.

**Results:**

Thirty patients were recruited and were assessed in the per protocol analysis. (IBZ: n=16; VAN n=14). Fifteen of 16 (93.8%) patients given IBZ had a clinical cure versus 14 of 14 (100%) patients given VAN. IBZ and VAN were well tolerated with no serious drug-related events. *C. difficile* was eradicated from all stool samples after day 3 of treatment for IBZ-treated patients while three VAN-treated patients had *C. difficile* growth after day 3. IBZ demonstrated consistent preservation of key Bacillota species while significant decreases were observed in VAN-treated patients. CDI recurrence occurred in two patients given VAN and no patients given IBZ.

**Conclusion:**

In the phase 2b study, IBZ had a clinically comparable cure rate and safety profile to oral vancomycin. Favorable microbiologic and microbiome findings were observed with IBZ-treated patients along with a lower rate of CDI recurrence. These results warrant further development in phase 3 trials.

**Disclosures:**

**Michael Silverman, MD**, Acurx: Advisor/Consultant **Kevin W. Garey, PharmD, MS**, Acurx: Grant/Research Support

